# Atheroma of the Left Coronary Artery, Resulting in Aneurism of the Apex of the Left Ventricle

**Published:** 1887-11

**Authors:** Robert Tilley

**Affiliations:** Chicago, Ill.


### ATHEROMA OF THE LEFT CORONARY ARTERY RESULTING IN ANEURISM OF THE APEX OF THE LEFT VENTRICLE.*

BY ROBERT TILLEY, M. D., OF CHICAGO.

As this subject is so little associated with my usual practice, it is with some hesitancy that I present even this short report. Aneurisms of the heart, however, are not common in clinical reports, and I trust that fact alone will justify the presentation of the case.

The patient was an intimate friend of mine, and it was only at his special request that I took charge of the case. He was 57 years of age, and his occupation was that of a lawyer. His previous life was unexceptionally exemplary. He did not use tobacco in any form, and was exceedingly temperate and method-

* Read before the Chicago Medical Society, June 6, 1887.

ical in all his habits; his carriage was such as to suggest the impossibility of any hurry on his part. His general build may be characterized as corpulent, although when young he was very thin. At the age of 30 he was declared to be dying of consumption. Recovery, however, seems to have occurred without medical assistance.

An ill-defined malaise, extending over a period of six weeks or two months, suddenly increased to such an extent on the 20th of October, 1885, that he was unable to leave the house, and was obliged to call assistance. This malaise consisted of wandering pains, not severe, over the chest, extending to the left shoulder and down the left arm, sometimes reaching the wrist. They were not periodic, and could not be associated with any definite act of daily life, but would rather come on when any change of action was about to take place.

He first called my attention to this thus: "I don't know whether I want to follow a doctor's directions or not. I get occasionally flying pains over my chest and in my left shoulder, but on walking about a little I can make them disappear." As he always had a marked antipathy for medicine of any kind, and I did not possess any firm conviction of any greater benefit likely to follow anything I could suggest than the benefit he claimed from exercise, I told him to continue to use the method he had found successful and report later. At this time I had no conception of the existence in his case of atheromatous coronary arteries, nor did I suspect that the wandering pains were associated with incipient angina pectoris.

About six weeks after the interview above referred to, an acute attack of difficulty of breathing, associated with severe coughing and anxiety of countenance, came on. From this time he did not leave the house only for short walks. The pulse at this time was 120, feeble, but regular; breathing very laborious; could not lie down on the back or left side, and only for a short time on the right side; cough was very troublesome; no special features present in the alimentary canal. On percussion, no perceptible enlargement of the heart was detected; percussion also failed to reveal any enlargement of the liver. On auscultation, the heart revealed no definite abnormal sounds. The principal feature which I observed was that of a systole; the ventricles seemed to stop as though shutting down on a pledget of wool. I find that Constantin Paul refers to this symptom very definitely as associated with aneurism, or what he calls false aneurism of the heart. It certainly struck me as the one striking feature of the case. There was at this time no œdema, no fever, no albumin in the urine.

From the beginning his condition was considered grave, and consultation was obtained from the first.

Drs. H. A. Johnson and R. H. Babcock both saw the case, and the former remained as consulting physician to the end. The various heart tonics, such as strychnia, arsenic, and digitalis, were used with no demonstrable effect except this, that when the digitalis was increased so as to diminish the frequency of the heart's action, the action became so tumultuous and incoördinate that it was deemed best to let it beat at the gait that it found most convenient. In about a fortnight after the commencement of the acute attack, anginal pains became more prominent, and atheroma of the coronary arteries was suspected. There was no evidence, however, of any atheroma of any superficial vessels. From this date a bottle containing carbonate of ammonia and camphor became his companion, supplemented with small pellets of nitroglycerine, 1-100 of a grain. The latter gave more satisfaction than the nitrate of amyl. The anginal pains were not at any time characteristic for their severity, but the pallor of countenance and anxious expression were characteristic. Later on the feature of a systole was not so well marked. The feet had exhibited, on several occasions, a tendency to œdema, but about ten days before death it began to increase rapidly, and no remedies were found capable of removing it. The œdema extended gradually to the hips, abdomen and chest, and about nine weeks after the acute attacks, while walking across the room, he fell dead.

I would like to remark here that he derived marked comfort from gentle exercise with fresh air. When the sidewalks were covered with ice so that he could not possibly walk out of doors, he would wrap up and walk in a room with the windows wide open. Nitro-glycerine and carbonate of ammonia were the only remedies that gave him any appreciable relief.

The autopsy, which he himself requested—he had a horror of being buried alive—reveals nothing remarkably peculiar except the condition of the heart. This autopsy was performed on the day following the death, by Dr. Frank Cary, in the presence of Drs. H. A. and Frank S. Johnson, R. H. Babcock and myself. The heart was somewhat enlarged, all the valves were competent, there was little or no atheroma manifest except in the coronary arteries. One of these I show you almost completely occluded. It was just below this manifest atheroma of the left coronary that in the wall of the left ventricle near the apex the aneurism had developed. I should characterize it as about the size of a large walnut. The wall of the heart in the thinnest part was estimated by Dr. F. Johnson as only two millimetres thick. The aneurismal cavity was filled with blood, part of which showed signs of organization and part signs of disintegration. The clot was evidently formed at two different periods. The microscopic sections, for which I am indebted to the kindness of Dr. F. S. Johnson, shows this well. There were no signs of chronic endocarditis, but some of slight chronic myocarditis.

Bramwell divides aneurisms of the heart into acute and chronic. Constantin Paul calls such cases false aneurisms, and then adopts the same division. "Acute aneurism," says Bramwell,[1] "may result from anything which causes rapid local softening of a limited portion of the eardiac wall. Acute ulcerative endocarditis, acute localized myocarditis and acute softening, the result of thrombosis of the coronory arteries, are the conditions which are most likely to cause acute local dilatation of this description."

"Chronic aneurisms of the heart," he continues, "are almost always the result of chronic myocarditis. Fatty degeneration seems to be an occasional though extremely rare cause of this condition." He nowhere calls direct attention to an atheromatous condition of the coronary arteries as a cause. Constantin Paul speaks of a case under his observation where there was an

[1]Bramwell, Diseases of the Heart, p. 576.

atheromatous condition of the aorta and he supposed this to be the starting point in the case that he reports.

Dr. Mary Putnam Jacobi says (in Wood's Reference Handbook): "The essential cause of heart aneurism is thus identical with arterial aneurism; the lesion of structure is, however, different, as might be expected from the difference of tissue in the heart and arteries. In the latter, that is, the arteries, non-traumatic aneurism nearly always depends on atheroma; in the former upon fibroid disease the result of chronic interstitial myocarditis.

In the present case, I think, there is no doubt that the starting point was the diminished calibre of the left coronary artery by this atheromatous condition diminishing the supply of nutriment to the corresponding tissue and thus producing the extreme thinness of the walls in the part supplied by the artery. The clot of blood was probably the result of the incapacity of the left ventricle to completely contract and expel its contents and probably occurred in part at the time of his acute sickness about nine weeks before death.

The presence of this clot of blood fully explained the characteristic sound which I clearly recognized at the first and which I described as though the ventricle contracted on a pledget of wood.

